# Pectoralis major muscle index as an opportunistic predictor of mortality in acute stroke patients treated with intravenous thrombolysis

**DOI:** 10.1007/s10072-025-08026-9

**Published:** 2025-02-20

**Authors:** Ezgi Yilmaz, Isa Furkan Sarier, Rahsan Gocmen, Ethem Murat Arsava, Mehmet Akif Topcuoglu

**Affiliations:** 1 Faculty of Medicine Hospital, Department of Neurology, Hacettepe University, Ankara, Turkey; 2 Faculty of Medicine Hospital, Department of Radiology, Hacettepe University, Ankara, Turkey; 3 Department of Neurology, Hacettepe University School of Medicine, Altindag, Ankara, 06230 Turkey

**Keywords:** Sarcopenia, Computed tomography, Thrombolysis, Acute stroke, Prognosis, Mortality

## Abstract

**Background:**

Premorbid sarcopenia in acute stroke indicates poor prognosis. Since formal sarcopenia tests cannot be performed, the muscle features imaged in diagnostic studies are opportunistically used as surrogates for sarcopenia in the acute period.

**Methods:**

In 110 consecutive acute ischemic anterior circulation stroke patients treated with intravenous tissue plasminogen activator alone (mean age: 73±13 years, 55% women), the cross-sectional area (CSA) and attenuation of pectoralis major and minor muscles and mediastinal adipose tissue were measured at admission computed tomography (CT) angiography source images.

**Results:**

Pectoralis major and minor muscle CSA (mm^2^) and indices (CSA/height(m)^2^) were significantly higher in patients with 3-month modified Rankin’s scores of 0–1 (excellent outcome, 41%), 0–2 (good outcome, 54%), and in surviving patients (87%). In regression models adjusted for age and NIHSS, pectoralis major muscle CSA (partial r: -0.281, *p* = 0.027) and pectoralis major index (partial r: -0.332, *p* = 0.008) were independent predictors of mortality. The discriminatory value of the pectoralis major index for mortality was good (ROC-AUC 0.794, 95%CI: 0.676–0.885). The optimal threshold for survival of pectoralis major index was > 3316 mm^2^/m^2^ with 0.607 Youden J index. No difference was found in muscle CT attenuation values, mediastinal adipose tissue area and radiodensity in deceased patients.

**Conclusions:**

Our retrospective analysis documents that the pectoralis major index, a readily available CT anthropometry surrogate for sarcopenia, is an independent predictor of survival in patients with acute ischemic stroke undergoing systemic thrombolysis. It may suggest that the pectoralis major index could be included in the prognostic toolkit of acute ischemic stroke.

**Supplementary Information:**

The online version contains supplementary material available at 10.1007/s10072-025-08026-9.

## Introduction

In patients with acute ischemic stroke, the frequency of preexisting sarcopenia is heterogeneous, but has been reported to be as high as 50% on average [1, 2]. Sarcopenia contributes to poorer clinical functional outcomes of acute stroke [3]. Preexisting sarcopenia worsens during the post-stroke period, as the stroke itself is a definite cause of limb muscle loss and is not limited to body parts with motor deficits [4].

In acute stroke patients, especially those with severe clinical conditions, it is often not possible to make a formal sarcopenia assessment due to obstacles such as cognition and mobility problems [5]. Therefore, the assessment of muscle size (or mass), one of the components of sarcopenia, has become more of a research tool than performance and function. For this purpose, articles investigating the importance of muscle thickness, cross-sectional area, volume and quality (e.g. computed tomography-based muscle tissue density) in the area of interest in readily-available neuroimaging studies obtained during the acute diagnosis process are frequently encountered. This opportunistic approach is of course a strategy frequently used not only in the setting of acute stroke, but also in other diseases such as oncological ones. For this purpose, craniocervical musculature measurements such as temporal and masseter muscles were frequently used in stroke literature [6, 7]. These opportunistic CT-based craniocervical sarcopenia markers were shown to modify clinical outcomes in patients who underwent mechanical thrombectomy and/or systemic thrombolytic therapy for their acute ischemic stroke [6–8].

We examined the imaging characteristics of the pectoral muscles and mediastinal adipose tissue on CT angiography source images to determine their effect on the efficacy of intravenous (IV) thrombolytic therapy in patients with acute ischemic stroke.

## Patients and methods

A total of 110 acute ischemic anterior circulation stroke patients (mean age: 73±13 years, 55% female) treated with IV tissue plasminogen activator (tPA) in the last ten years were retrospectively extracted from the Hacettepe University Departmental acute stroke database and evaluated for this analysis. A total of 104 (48%) patients were excluded from the study. 39 patients were excluded because of insufficient quality CT angiography images or missing pectoral and mediastinal slices for analysis (see below). Additionally, 42 patients with recurrent stroke (16 with modified Rankin’s score 3 or higher), 13 patients with a history of cancer, 8 patients with gait disturbance due to orthopedic, rheumatologic, or neurologic causes, and 2 patients received lytic therapy at an outside hospital were excluded. The clinicoradiologic characteristics of these patients did not differ from those included (Data not shown). In addition, 2 patients with missing 3-month prognosis information were excluded. Of note, patients who underwent interventional treatment alone or in addition to IV tPA were excluded. The database and its protocols were approved by the University’s non-interventional ethics committee (Decision no: 2022/07–48). Details of the features in the database, such as patient registration, etiological investigation protocol, and stroke classification system, can be found elsewhere [9, 10]. Briefly, the “Causative Classification System” was used to classify etiological Stroke subgroups [11]. The National Institutes of Health Stroke Scale (NIHSS) was determined before IV tPA treatment, at twenty-four hours and at discharge [12]. Modified Rankin scale (mRS) information was collected at three months after stroke [13].

A “positive” effect of IV tPA was defined as a decrease of four or more points in the NIHSS total score or a decrease of the total score to zero within 24 h. Response to IV tPA was categorized as “dramatic” if there was a decrease of 8 or more in the NIHSS score or if the score was reduced to 1 or 0 [14]. Functional outcome was classified as “good/positive” if the 90-day mRS was 2 or less and “favorable/excellent” if the mRS was 1 or 0. Fiorelli’s parenchymal hemorrhage type-2 (PH-2, symptomatic) and any hemorrhagic transformation after IV tPA were assessed on 24-hour brain CT follow-up [15].

### Image analysis protocol

Analyses were performed on CT angiography source images obtained within the first 24 h of admission. CT angiograms were acquired with multidetector devices (SOMATOM emotion Duo, Sensation 16 or Perspective with 64 slice configuration; Siemens, Erlangen, Germany). The imaging parameters of CTA were a tube voltage of 120 kV, aortic arch to apex, 100 mAs, slice width 1 mm, and slice collimation 75 mm. For timing the spiral scan and acquisition, a dynamic contrast bolus detection technique (CareBolus; Siemens Medical Systems, Erlangen, Germany) was employed. This technique involved administering a single bolus of approximately 100 mL of nonionic contrast medium with an initial delay of 3–4 s into the antecubital vein. The analysis of the pectoral muscles and mediastinal fat tissue was performed using the section at the level of the tracheal carina.

After the interobserver reliability analysis conducted at the beginning of the study, the images were evaluated by the study neurologists (IFS, EY), who were blinded to the clinical data (Condordance correlation coefficients between 0.8209 and 0.9503, see Supplementary Table 1 for detais). The study’s senior neuroradiologist (RG) provided training on parameter measurement and optimization, and resolved any inconsistencies that arose. The cross-sectional area (mm^2^) and mean attenuation (HU) of the pectoral muscles (pectoralis major and minor muscles) and mediastinal fat tissue were evaluated. The indices were calculated by dividing the cross-sectional areas by the square of the heights.

**Fig. 1:**
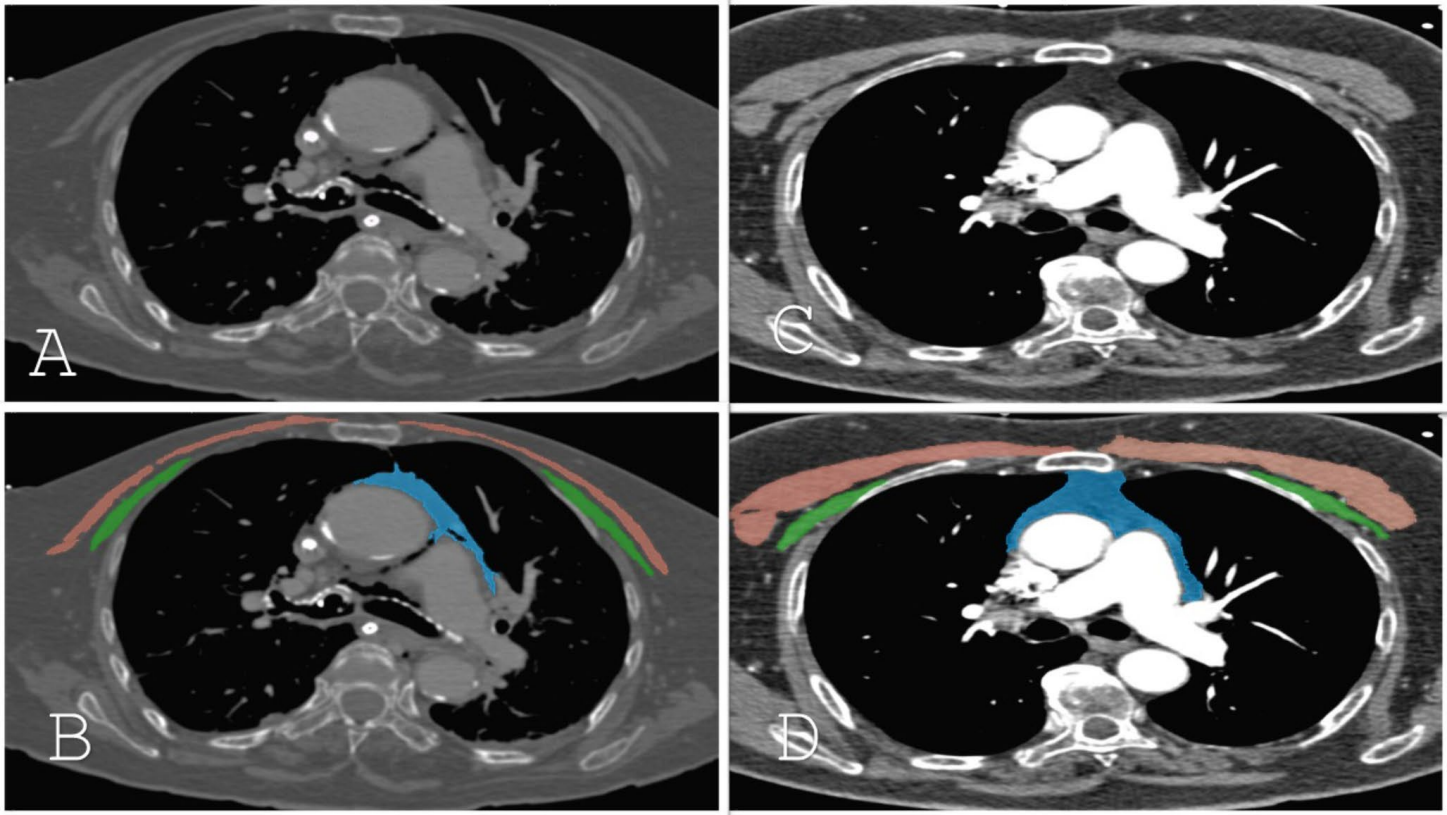
Axial CTA images and segmented areas of pectoral muscles and mediastinal fat tissues at the tracheal carina level for two different patients (**A-D**). Segmented CTA images illustrating the anatomical structures with distinct colors: the pectoralis major muscle is shown in red, the pectoralis minor muscle in green, and the mediastinal fat tissue in blue (**B, D**). Patient 1 (**A, B**): Mean Pectoralis Major Area: 3808 mm^2^, Mean Pectoralis Major Density: 26 HU, Mediastinal Fat Tissue Area: 3998 mm^2^, Mean Mediastinal Fat Tissue Density: -79 HU. Patient 2 (**C, D**): Mean Pectoralis Major Area: 13,746 mm^2^, Mean Pectoralis Major Density: 41 HU, Mediastinal Fat Tissue Area: 6215 mm^2^, Mean Mediastinal Fat Tissue Density: − 68 HU

Measurements were performed using a semi-automatic segmentation method with publicly available image processing software. (ImageJ version 1.54f, National Institutes of Health, USA) [16] After manually marking the pectoral muscles and mediastinal fat tissue boundaries, the thresholds were set to -150 to -30 HU for the fat tissue and − 29 to 150 for the muscle [17–19]. (Fig. 1) Since measurements were performed on imaging acquired for angiographic purposes, some images had incomplete coverage of the pectoral muscle area, particularly on the left side. Consequently, we excluded any muscles with unclear boundaries from the analysis. For muscle parameters, if bilateral assessment was possible, we included the average measurements from both sides; otherwise, only the measurements from the evaluated side were included in the analysis.

## Statistics

All data are presented as mean ± standard deviation or percentage as appropriate. Normality of distribution was examined by histogram and QQ plot visual analysis, Shapiro-Wilk’s and Kolmogorov-Smirnov tests. Student’s t and Mann Whitney U were used for numerical values and chi-square or Fisher’s exact tests were used for categorical variables. Possible relationships were tested in multiple exploratory linear regression models. Separate models were created for testing the relationship between functional outcomes, symptomatic hemorrhagic transformation and mortality after IV thrombolytic treatment and pectoralis major and minor cross-sectional areas along with indexes as opportunistic sarcopenia parameters. Adjustments were performed for age and NIHSS. Partial-r and p values of the models were reported. Receiver operating characteristic (ROC) analysis was performed for the parameters found to be positive and the area under the ROC curve (AUC) was calculated. The mean of the ROC-AUC value was reported with standart errors and 95% confidence interval (CI). AUC values between 0.5 and 0.6 are interpreted as “unsatisfactory”; 0.6 to 0.7 as “satisfactory”, 0.7 to 0.8 as “good”, 0.8 to 0.9 as “very good”, and greater than 0.9 as “excellent”. The most appropriate threshold value was determined with Youden statistics and the sensitivity and specificity values along with their 95% CI and positive and negative likelihood ratios (+ LR and -LR) of this value were reported. Youden index acceptability level was determined as 50%. Lin’s concordance correlation coefficient analysis was used to test inter-rater reliability of CT measures; and correlation coefficient and its 95%CIs were reported. A p value of less than 0.05 was accepted as statistical significance criterion. Statistical Package for Social Sciences (SPSS) version 22.0 (SPSS Inc., Chicago, Illinois, USA) program was used for all analyses.

## Results

Fourteen (13%) of acute ischemic stroke patients who received IV tPA died within 3 months. In univariate analysis, patients who died had significantly higher admission NIHSS (approximately 6 points at average, *p* < 0.001) and symptom-to-door-time (22.1 min longer at average, *p* = 0.029). Although patients who died had a higher vascular risk factor frequency, this was not statistically significant (Table-1). Compared with survivors, patients who died had significantly lower pectoralis major cross-sectional muscle area (mean 35.2 cm^2^ and 69.2% less than survivors, *p* = 0.012) and pectoralis major index (mean 13.2 cm^2^/m^2^ and 68.9% less than survivors, *p* = 0.004). Similarly, pectoralis minor cross-sectional muscle area (mean 8.7 cm^2^ and 76.8% less than survivors, *p* = 0.048) and pectoralis minor index (mean 2.88 cm^2^/m^2^ and 78.9% less than survivors, *p* = 0.046) were significantly lower. No difference was found in muscle CT attenuation values, mediastinal adipose tissue area and radiodensity in deceased patients (Table-1). In regression models adjusted for age and NIHSS, pectoralis major muscle size (partial r for cross-sectional area: -0.281 (*p* = 0.027) and pectoralis major index − 0.332 (*p* = 0.008) were independent predictors of mortality. Pectoralis minor muscle size (partial r for cross-sectional area: -0.164 (*p* = 0.073) and pectoralis minor index − 0.189 (*p* = 0.051) tended to be marginal, but not significant, predictors of mortality (Supplementary Web Table 1).

**Table 1:** Prognosis and muscle/adipose tissue parameters

		Expired	Survived		mRS 0–1	mRS 2–6		mRS 0–2	mRS 3–6	
n		14	96	p	45	65	P	59	51	p
Age		77,3 ± 11,6	72,6 ± 13,5	0.216	66.3 ± 14.6	73.4 ± 12.4	< 0.001	69.1 ± 14.6	77.9 ± 9.9	< 0.001
Female		64%	54%	0.477	51%	59%	0.446	51%	61%	0.296
Body Mass index (kg/m^2^)		25,8 ± 5,5	26,6 ± 5	0.596	27.9 ± 5.4	26.8 ± 5	0.070	27.1 ± 5.5	25.7 ± 4.3	0.126
Hypertension		93%	71%	0.081	64%	80%	0.690	66%	82%	0.054
Diabetes mellitus		50%	28%	0.098	22%	37%	0.101	24%	39%	0.080
Dyslipidemia		21%	23%	0.901	18%	26%	0.303	205	26%	0.520
Atrial fibrillation		57%	38%	0.161	31%	46%	0.113	32%	49%	0.073
Active smoking		14%	24%	0.420	29%	19%	0.199	25%	20%	0.468
NIHSS admission		17,8 ± 6,2	11,7 ± 5,6	< 0.001	9.1 ± 5.5	14.2 ± 6.5	< 0.001	9.8 ± 5	15.5 ± 5.6	< 0.001
Symptom-to-door [minutes]		64,8 ± 39,9	84,7 ± 48,2	0.145	81.4 ± 57.5	89.4 ± 98.4	0.453	77.9 ± 44	87 ± 51.4	0.320
Door-to-CT [minutes]		17,3 ± 7,4	21,4 ± 13,1	0.279	21.5 ± 11.8	20.3 ± 11.9	0.430	21.9 ± 12.6	19.7 ± 12.6	0.355
Door-to-needle [minutes]		119,3 ± 38,7	97,2 ± 33	0.029	91.3 ± 33.6	101.3 ± 44.5	0.056	94.6 ± 33.3	106.5 ± 34.9	0.080
Length of stay (days)		26,6 ± 20,4	16 ± 22	0.092	7.1 ± 6.2	20 ± 24.9	< 0.001	9.1 ± 13.1	26.8 ± 26.1	< 0.001
Pectoralis major	Cross sectional area (mm^2^)	7889 ± 2969,4	11407,8 ± 4480,6	0.012	12564.2 ± 4562.5	10185.5 ± 4187.9	0.047	12266.8 ± 4584.9	9708.8 ± 4077.1	0.022
	Cross sectional area/height^2^	2919,4 ± 852,5	4238 ± 1480	0.004	4660.6 ± 1501.6	3710 ± 1382	0.018	4551.7 ± 1500.2	3607 ± 1341.9	0.011
	Average density [HU]	52,5 ± 25,1	48,3 ± 13,8	0.425	52.3 ± 12	47.5 ± 8	0.280	49.8 ± 12.1	48.6 ± 18.9	0.782
	Median density [HU]	57,1 ± 26,3	52 ± 16,1	0.394	56.1 ± 14.2	51.7 ± 19.3	0.382	53.3 ± 13.8	52.7 ± 21.1	0.897
	Density standard deviation	30,1 ± 6,1	30,2 ± 4,2	0.942	30.8 ± 4.6	30.2 ± 4.5	0.606	29.6 ± 4.2	30.6 ± 4.9	0.426
Pectoralis minor	Cross sectional area (mm^2^)	2889,9 ± 1032,4	3763,8 ± 1586,4	0.048	4123.8 ± 1832.7	3316.4 ± 1255.1	0.006	3949 ± 1770.8	3309.7 ± 1176.5	0.030
	Cross sectional area/height^2^	1075,9 ± 395,9	1364,4 ± 511,5	0.046	1464.5 ± 607.4	1232.9 ± 400.2	0.018	1412.1 ± 582.4	1230 ± 382.8	0.059
	Average density [HU]	46,5 ± 22,2	45,2 ± 12,5	0.753	46 ± 14.4	44.1 ± 14.2	0.485	45.5 ± 12.1	45.3 ± 16	0.925
	Median density [HU]	48,4 ± 24,4	48,7 ± 14,4	0.935	49.7 ± 16.1	47.3 ± 16.1	0.422	49.1 ± 13.9	48.2 ± 18.1	0.758
	Density standard deviation	31,9 ± 7,5	31,9 ± 5,5	0.987	31.9 ± 5.5	32.4 ± 5.9	0.626	31.5 ± 5.4	32.3 ± 6.2	0.486
Mediastinal adipose tissue	Cross sectional area (mm^2^)	9407,5 ± 10360,5	9283,2 ± 6551,7	0.951	9237.9 ± 6597.9	9326.4 ± 7225.6	0.938	9888.9 ± 7305	8616.5 ± 6832.4	0.350
	Cross sectional area/height^2^	3405,6 ± 3616,3	3390,1 ± 2258	0.983	3381.7 ± 2352.8	3381.7 ± 2352.8	0.835	3575.9 ± 2532.4	3179.5 ± 2358.6	0.400
	Average density [HU]	-80 ± 9,6	-83,4 ± 8,5	0.175	-82.4 ± 9.2	-82.9 ± 8.7	0.378	-82.6 ± 8.8	-83.3 ± 8.4	0.713
	Median density [HU]	-79,3 ± 12,5	-82,6 ± 10,8	0.299	-81.0 ± 11.5	-82.9 ± 10.7	0.375	-81.6 ± 11.1	-82.7 ± 11	0.609
	Density standard deviation	28,5 ± 2,7	29,1 ± 2,6	0.400	29.5 ± 2.7	29.2 ± 2.4	0.481	29.1 ± 2.6	29 ± 2.6	0.865

The ROC-AUC of pectoralis major cross-sectional area for the discrimination of survival was “good” [AUC = 0.757±0.0878 (95% CI: 0.638 to 0.851, *p* < 0.001)]; and the optimum threshold was found to be 9769 mm^2^. The sensitivity of this value was 91.67% (95% CI: 61.5-99.8%), specificity was 55.17% (95% CI: 41.5-68.3%), +LR was 2.04 and–LR was 0.15. The Youden J index was found to be 0.468 (marginally effective if not ineffective). The discrimination value of pectoralis major index for death was “almost very good” [ROC-AUC = 0.794±0.0637 (95%CI: 0.676 to 0.884, *p* = 0.001). The optimum threshold of pectoralis major index for survival was > 3316 mm^2^/m^2^ and its sensitivity was 83.33% (95%CI: 51.6 − 97.9%), specificity was 77.36% (95%CI: 63.8 − 87.7%), +LR was 3.68 and–LR was 0.22. Youden J index was found to be 0.6069 (that is, “effective”).

At the third month, 41% (*n* = 45) of the cases had excellent (mRS 0–1) functional results. The mean age and NIHSS of these patients were significantly lower than those without, and the duration of hospital stay was also significantly shorter. Pectoralis major and minor muscle cross-sectional area and indices were significantly higher (Table-1). Pectoral muscle radiodensity values were not different between the groups. When adjusted for age and NIHSS in linear regression models, the correlation between excellent functional results and pectoralis major muscle area and index (partial correlation coefficients 15.3% and 19.7%, respectively) and pectoralis minor area and index (partial correlation coefficients 7.4% and 9.1%, respectively) did not survive at significance level (Table-1; Supplementary web Table 2). However, the pectoralis major cross sectional area and pectoralis major index increased to a significant predictive level for favorable prognosis (partial-r and p values: *r* = 0.313, *p* = 0.014 and *r* = 0.301, *p* = 0.018, respectively) by exploratively adding female gender, which was not significant but numerically higher in the univariate analysis, to the regression model. The average mediastinal fat tissue area, indices and attenuation values in the patients with very good clinical results were not different from the others (Table-1).

Positive clinical outcome (mRS 0–2) was achieved in 59 (54%) patients. The mean age and admission NIHSS scores of these patients were significantly lower. While the duration of hospital stay was shorter, no difference was observed in terms of risk factors and stroke etiology. In univariate analysis, it was determined that the pectoralis major muscle area increased significantly with good clinical outcome (difference between areas was approximately 25.6 cm^2^ and difference between indices was 9.4 cm^2^/m^2^) and the pectoralis minor cross-sectional area and index showed a strong trend (average area was 6.4 cm^2^ and index was 1.82 cm^2^/m^2^ higher) (Table-1). However, in regression models adjusted for age and NIHSS, the relationship between pectoral muscle area (partial r: 16.5% for major and 6.1% for minor) and index (partial r: 20.3% for major and 5.3% for minor) and the chance of mRS 0–2 outcome did not remain significant (Supplementary web Table 2). With the addition of female gender as an independent variable in this model exploratively, the pectoralis major cross-sectional area (partial-*r* = 0.280, *p* = 0.029) and the pectoralis major index (partial-*r* = 0.275, *p* = 0.032) have reached statistically significant levels for predicting a good prognosis. There was no difference between patients with positive and negative outcomes in terms of attenuation values of pectoral muscles and mediastinal adipose tissue area, index and CT densities (Table-1).

At 24 h, 47% of patients responded favorably to thrombolytic therapy and 23% responded excellently. There were no statistically significant differences in muscle and adipose tissue CT features between tPA responders and non-responders. Only in cases with positive response to IV tPA, pectoralis major cross sectional area and pectoralis major index were found to be borderline higher [Cross-sectional area: 11855.7 ± 4620.7 vs. 9939 ± 4118.3, mm^2^, *p* = 0.082; and pectoralis major index 4392.2 ± 1592.3 vs. 3693.5 ± 1295, mm^2^/m^2^, *p* = 0.055, see Supplementary web Table 3 for details). Symptomatic tPA-related hemorrhage (Parenchymal hemorrhage type 2) was observed in 3 patients (2.7%) and no significant differences were noted in tomographic muscle and adipose tissue characteristics in these patients (see Supplementary web Table 4 for details).

## Discussion

We herein report the effects of cross-sectional area and radiodensity of the pectoral muscles and mediastinal fat tissue, which we propose as readily available opportunistic sarcopenia and sarcopenic obesity CT markers, obtained on admission craniocervical CT-angiography source images, on clinical outcomes in acute stroke patients treated with IV tPA. We documented that reduced pectoral muscles cross-sectional area and index is an independent predictor of mortality and may be associated with poor prognosis in these patients. In addition, we found that the effect of thrombolytic therapy was not modified by mediastinal fat tissue CT parameters in acute stroke patients.

It has been determined that muscle parameters in clinical imaging performed for the main disease diagnosis and follow-up provide important insight into the prognosis of many oncological and non-oncological diseases [20]. In this context, the masseter and temporalis muscles have been studied the most in acute stroke setting [21]. It is generally accepted that temporal muscle thickness or masseter thickness and area represent premorbid sarcopenia in stroke patients [22]. However, since both muscles are masticatory muscles that opening the jaw, it is not expected to have a direct specific negative effect on disability and locomotor function loss caused by stroke, except for post-stroke dysphagia [23, 24].

The main function of the pectoralis major muscle is adduction and internal rotation of the arm in the shoulder joint. In addition, its clavicular part helps to flex the extended arm up to 90° and its sternocostal part facilitates the extension of the flexed arm by pulling it downwards. The pectoral minor muscle stabilizes the scapula by drawing it inferiorly and anteriorly against the thoracic wall. In addition, both are accessory respiratory muscles. Therefore, measuring the pectoral muscles on CT angiography source images may be a more convenient option than the masticatory muscles in the context of measuring the effectiveness of acute treatments, as it reflects the return of upper extremity function after stroke. In addition, it has been documented in the oncology literature that the pectoral muscles are reliable prognostic predictors, especially in patients with breast and lung cancer, and that they are a muscle group that represents general sarcopenia well and objectively [25, 26]. However, since this muscle group is not imaged in brain CT obtained in the setting of acute stroke, its examination as an opportunistic sarcopenia marker has not been done until our study. As we documented in our study, it is possible to examine this muscle group in most patients on craniocervical CT angiography source images.

Our study found that the pectoralis major index may help predict survival outcomes in acute stroke patients before IV tPA administration, showing potential to discriminate and stratify survival risk. A pectoralis major index above 33 cm^2^/m^2^ provides a potentially useful clinical yield for survival (mean ROC-AUC = 0.794, 95%CI lower limit of ROC-AUC 0.676, Youden J index 0.6069, that is, “effective”). Based on these data, we suggest that the pectoralis major index, which has a prognostic value since it is readily available in the acute phase of stroke, be used for practical detection of pre-morbid sarcopenia. Pectoralis minor muscle area and indexes showed a similar trend, although they did not reach a statistically significant level in multivariate analysis. Pectoral muscle CT parameters in patients receiving IV tPA in acute stroke have not been reported before. However, in a previous study, the effect of pectoral muscle area and attenuation on prognosis and survival in patients undergoing mechanical thrombectomy for anterior circulation ischemic stroke was investigated by directly measuring the thoracic CT obtained for this purpose, i.e., not opportunistically as in our study [19]. They showed that the cross-sectional area of the pectoralis major CT area was not found to be a significant predictor of 90-day mortality in acute stroke patients undergoing interventional treatment, unlike our study. However, pectoralis muscle radiodensity was found to be an independent risk factor (OR 0.896) for 90-day mortality. We did not find that muscle CT attenuation, which is suggested as an indicator of myosteatosis, had a significant effect in changing the effect of tPA in acute stroke. The differences between this study and our findings may be due to differences in study designs, treatment techniques, imaging methods, and imaging timing, and this should certainly be the subject of further studies. However, since no additional imaging is performed in our model, no extra time loss is expected in the hyper-acute stroke in-hospital period, where very rapid action must be taken before IV tPA, and has the potential to be a cost-effective approach.

The retrospective nature of our study, which was performed from a prospectively gathered database, and the relatively small number of patients (for example, the frequency of bleeding associated with tPA was quite rare) should be noted as limitations. We emphasize that the pectoral muscles reflect upper limb function, but again due to the retrospective nature of the study we were not able to measure or score this directly. Additionally, we were unable to correlate pectoralis muscle measurements with established systemic sarcopenia detection methods such as bioelectrical impedance. And, since our study was limited to patients who received only IV thrombolytic therapy, the results cannot be generalized to those received conservative treatment or mechanical thrombectomy. All of these may be the subject of future research.

In conclusion, we propose the pectoralis major index as a readily available CT anthropometric surrogate for premorbid sarcopenia in acute stroke patients. Given the importance of the pectoralis muscle in locomotor function, the pectoralis index may be added to the neurologist’s prognostic toolkit in the acute stroke thrombolysis setting.

## Electronic supplementary material

Below is the link to the electronic supplementary material.

Supplementary Material 1

## Abbreviations

CI: Confidence interval
CSA: Cross-sectional area
CTA: Computed tomography angiography
IV tPA: Intravenous tissue plasminogen activator
mRS: Modified Rankin scale
NIHSS: National Institutes of Health Stroke Scale
PH-2: Parenchymal hemorrhage type-2
ROC-AUC: Area under the Receiver operating characteristic (ROC) curve

## Declarations

### Guarantor

MAT.
