# Supplementary material for: Pectoralis major muscle index as an opportunistic predictor of mortality in acute stroke patients treated with intravenous thrombolysis
Source: Neurol Sci. 2025 Feb 20;46(5):2195–202. doi: 10.1007/s10072-025-08026-9 (PMC12003620; doi:10.1007/s10072-025-08026-9)
Supplement: Supplementary file 1 — Supplementary Material 1 [file 10072_2025_8026_MOESM1_ESM.docx]

**Supplementary Web Tables**

**Supplementary Web Table 1:** Concordance correlation analysis Results

|  |  | Concordance correlation coefficient | 95% Confidence interval | Pearson ρ (precision) | Bias correction factor Cb (accuracy) |
| --- | --- | --- | --- | --- | --- |
| Pectoralis major | Cross sectional area (mm^2^) | 0,9481 | 0,8590 to 0,9815 | 0,9854 | 0,9622 |
|  | Average density [HU] | 0,9504 | 0,8402 to 0,9852 | 0,9698 | 0,9800 |
|  | Median density [HU] | 0,9871 | 0,9586 to 0,9960 | 0,9945 | 0,9926 |
|  | Density standard deviation | 0,7868 | 0,5094 to 0,9161 | 0,9372 | 0,8396 |
| Pectoralis minor | Cross sectional area (mm^2^) | 0,8209 | 0.6204 to 0.9207 | 0,8303 | 0,9887 |
|  | Average density [HU] | 0,9530 | 0,8801 to 0,9820 | 0,9656 | 0,9869 |
|  | Median density [HU] | 0,9532 | 0,8884 to 0,9808 | 0,9646 | 0,9883 |
|  | Density standard deviation | 0,6322 | 0,3193 to 0,8208 | 0,7934 | 0,7968 |
| Mediastinal adipose tissue | Cross sectional area (mm^2^) | 0,9503 | 0,8926 to 0,9774 | 0,9738 | 0,9758 |
|  | Average density [HU] | 0,9856 | 0,9644 to 0,9942 | 0,9861 | 0,9995 |
|  | Median density [HU] | 0,9572 | 0,8965 to 0,9826 | 0,9580 | 0,9991 |
|  | Density standard deviation | 0,9184 | 0,8103 to 0,9661 | 0,9218 | 0,9963 |

**Supplementary Web Table 2:** Multipl regression analysis results

|  | mRS 0-1 | | | | mRS 0-2 | | | | Mortality | | | |
| --- | --- | --- | --- | --- | --- | --- | --- | --- | --- | --- | --- | --- |
|  | Pectoralis major | | Pectoralis minor | | Pectoralis major | | Pectoralis minor | | Pectoralis major | | Pectoralis minor | |
|  | mm^2^ | mm^2^/m^2^ | mm^2^ | mm^2^/m^2^ | mm^2^ | mm^2^/m^2^ | mm^2^ | mm^2^/m^2^ | mm^2^ | mm^2^/m^2^ | mm^2^ | mm^2^/m^2^ |
| **r_partial_** | **0.153** | **0.197** | **0.074** | **0.091** | **0.165** | **0.203** | **0.061** | **0.053** | **-0.281** | **-0.332** | **-0.164** | **-0.189** |
| p | 0.235 | 0.124 | 0.447 | 0.351 | 0.199 | 0.114 | 0.531 | 0.589 | 0.027 | 0.008 | 0.073 | 0.051 |
| **Age-r_partial_** | **-0.170** | **-0.146** | **-0.162** | **-0.142** | **-0.087** | **-0.065** | **-0.120** | **-0.127** | **-0.100** | **-0.122** | **-0.109** | **-0.114** |
| p | 0.186 | 0.256 | 0.096 | 0.105 | 0.501 | 0.613 | 0.219 | 0.194 | 0.438 | 0.306 | 0.262 | 0.241 |
| **NIHSS-r_partial_** | **-0.105** | **-0.102** | **-0.304** | **-0.307** | **-0.236** | **-0.234** | **-0.400** | **-0.400** | **0.177** | **0.176** | **0.333** | **0.332** |
| P | 0.415 | 0.432 | 0.001 | 0.001 | 0.065 | 0.067 | <0.001 | 0.001 | 0.169 | 0.172 | <0.001 | <0.001 |

**Supplementary Web Table 3.** Thrombolysis effectiveness and muscle/adipose tissue parameters

|  | | Positive effect | | | Dramatic effect | | |
| --- | --- | --- | --- | --- | --- | --- | --- |
|  | | Yes | No |  | Yes | No |  |
| N | | 51 | 58 | p | 25 | 84 | p |
| Age | | 73,5 ± 14,7 | 72,9 ± 12,2 | 0.814 | 75,1 ± 13,9 | 72,6 ± 13,2 | 0.413 |
| Female | | 57% | 52% | 0.591 | 76% | 48% | 0.012 |
| Body Mass index (kg/m^2^) | | 26,4 ± 5,1 | 26,6 ± 5,1 | 0.845 | 27,6 ± 6,1 | 26,2 ± 4,7 | 0.236 |
| Hypertension | | 65% | 81% | 0.054 | 72% | 74% | 0.857 |
| Diabetes mellitus | | 28% | 35% | 0.547 | 28% | 31% | 0.778 |
| Dyslipidemia | | 16% | 31% | 0.061 | 16% | 26% | 0.294 |
| Atrial fibrillation | | 39% | 41% | 0.818 | 40% | 41% | 0.966 |
| Active smoking | | 24% | 22% | 0.890 | 125 | 26% | 0.138 |
| NIHSS admission | | 12,9 ± 5,1 | 12,2 ± 6,7 | 0.571 | 14,9 ± 4,6 | 11,8 ± 6,2 | 0.021 |
| Symptom-to-door [minutes] | | 74,8 ± 45,7 | 83,1 ± 40,5 | 0.318 | 77,2 ± 47,3 | 79,8 ± 42 | 0.791 |
| Door-to-CT [minutes] | | 19,1 ± 9,1 | 21,3 ± 14,2 | 0.340 | 19,4 ± 8,6 | 20,5 ± 12,9 | 0.695 |
| Door-to-needle [minutes] | | 91,6 ± 31,2 | 106,4 ± 35,9 | 0.027 | 92,8 ± 34,9 | 101,6 ± 34,3 | 0.270 |
| Length of stay (days) | | 11,9 ± 21,2 | 21,1 ± 19,9 | 0.021 | 8,3 ± 11,3 | 19,4 ± 22,5 | 0.020 |
| Pectoralis major | Cross sectional area (mm^2^) | 11855,7 ± 4620,7 | 9939 ± 4118,3 | 0.082 | 10525,8 ± 4366 | 10881,7 ± 4479,1 | 0.782 |
|  | Index* | 4392,2 ± 1592,3 | 3693,5 ± 1295 | 0.055 | 3937,8 ± 1425,3 | 4027,2 ± 1492,8 | 0.834 |
|  | Average density [HU] | 49,4 ± 13,4 | 48,6 ± 18,4 | 0.840 | 46,5 ± 15,9 | 49,8 ± 16,4 | 0.491 |
|  | Median density [HU] | 53,6 ± 16,2 | 52,1 ± 20 | 0.748 | 50,2 ± 19 | 53,7 ± 18,1 | 0.514 |
|  | Density standard deviation | 30,6 ± 3,9 | 29,8 ± 5,1 | 0.496 | 31,3 ± 3,6 | 29,8 ± 4,8 | 0.271 |
| Pectoralis minor | Cross sectional area (mm^2^) | 3770,4 ± 1568,9 | 3563,3 ± 1561,8 | 0.492 | 3686,1 ± 1948,1 | 3652,5 ± 1440,3 | 0.925 |
|  | Index* | 1366,3 ± 518,9 | 1296 ± 501,6 | 0.474 | 1368,9 ± 653,3 | 1317 ± 461,1 | 0.656 |
|  | Average density [HU] | 44,1 ± 12,2 | 45,9 ± 15,6 | 0.513 | 44,2 ± 13,2 | 45,3 ± 14,4 | 0.752 |
|  | Median density [HU] | 47,6 ± 14,1 | 48,7 ± 17,4 | 0.734 | 47,2 ± 16 | 48,5 ± 16 | 0.732 |
|  | Density standard deviation | 32,1 ± 5,6 | 31,6 ± 6 | 0.669 | 32,3 ± 5,5 | 31,7 ± 5,9 | 0.697 |
| Mediastinal adipose tissue | Cross sectional area (mm^2^) | 10021,7 ± 8488,2 | 8819,6 ± 5567,5 | 0.379 | 10568,4 ± 9532,9 | 9028,9 ± 6191,6 | 0.342 |
|  | Index* | 3621,1 ± 2889,2 | 3245,2 ± 1987,2 | 0.426 | 3897,2 ± 3266,2 | 3279,4 ± 2147,9 | 0.270 |
|  | Average density [HU] | -82,5 ± 8,4 | -83,4 ± 8,9 | 0.556 | -82,2 ± 8,3 | -83,2 ± 8,8 | 0.594 |
|  | Median density [HU] | -81,2 ± 10,9 | -83,1 ± 11,2 | 0.365 | -80,4 ± 10,5 | -82,7 ± 11,2 | 0.365 |
|  | Density standard deviation | 29 ± 2,8 | 29,1 ± 2,4 | 0.893 | 28,9 ± 3,1 | 29,1 ± 2,4 | 0.766 |

* Cross sectional area/height^2^ [mm^2^/m^2^]; Note: Information could not be obtained in one case.

**Abbreviations:** HU: Haunsfield’s unit NIHSS: The National Institutes of Health Stroke Scale, CT: Brain computed tomography

**Supplementary Web Table 4.** Parenchymal hemorrhage type 2 [symptomatic tPA-associated cerebral hemorrhages] and muscle/adipose tissue parameters

|  | | PH2 | | |
| --- | --- | --- | --- | --- |
|  | | Yes | No |  |
| n | | 3 | 107 | p |
| Age | | 72 ± 13 | 73,3 ± 13,3 | 0.863 |
| Female | | 100% | 53% | 0.108 |
| Body Mass index (kg/m^2^) | | 27,7 ± 6,3 | 26,4 ± 5 | 0.596 |
| Hypertension | | 100% | 73% | 0.389 |
| Diabetes mellitus | | 0% | 32% | 0.321 |
| Dyslipidemia | | 33% | 23% | 0.674 |
| Atrial fibrillation | | 67% | 29% | 0.343 |
| Active smoking | | 0% | 23% | 0.347 |
| NIHSS admission | | 15,7 ± 8,7 | 12,5 ± 5,9 | 0.363 |
| Symptom-to-door [minutes] | | 62,3 ± 4,9 | 82,2 ± 47,8 | 0.474 |
| Door-to-CT [minutes] | | 9,5 ± 2,1 | 21 ± 12,5 | 0.200 |
| Door-to-needle [minutes] | | 92,5 ± 6,4 | 99,7 ± 34,8 | 0.771 |
| Length of stay (days) | | 15,3 ± 13,7 | 17,6 ± 22,3 | 0.864 |
| Pectoralis major | Cross sectional area (mm^2^) | 7465 ± 3214 | 10955,2 ± 4425,9 | 0.184 |
|  | Cross sectional area/height^2^ | 2880,5 ± 1165,6 | 4059,6 ± 1464,5 | 0.176 |
|  | Average density [HU] | 44,7 ± 19 | 49,2 ± 16,2 | 0.643 |
|  | Median density [HU] | 49,7 ± 19,5 | 53 ± 18,3 | 0.763 |
|  | Density standard deviation | 30,3 ± 2,5 | 30,1 ± 4,6 | 0.945 |
| Pectoralis minor | Cross sectional area (mm^2^) | 2975,7 ± 575,4 | 3688,3 ± 1557,1 | 0.432 |
|  | Cross sectional area/height^2^ | 1155,8 ± 165,6 | 1338 ± 508,7 | 0.539 |
|  | Average density [HU] | 39,7 ± 18,5 | 45,5 ± 14 | 0.484 |
|  | Median density [HU] | 40,3 ± 23,5 | 48,9 ± 15,8 | 0.364 |
|  | Density standard deviation | 31,7 ± 5,9 | 31,8 ± 5,8 | 0.961 |
| Mediastinal adipose tissue | Cross sectional area (mm^2^) | 9595 ± 4847,5 | 9271,9 ± 7095 | 0.938 |
|  | Cross sectional area/height^2^ | 3685,4 ± 1754,7 | 3375,8 ± 2452,1 | 0.829 |
|  | Average density [HU] | -82 ± 4,4 | -83 ± 8,9 | 0.854 |
|  | Median density [HU] | -82,7 ± 4,6 | -82,1 ± 11,4 | 0.935 |
|  | Density standard deviation | 29 ± 1,7 | 29,1 ± 2,6 | 0.966 |

* Cross sectional area/height^2^ [mm^2^/m^2^]; Note: Information could not be obtained in one case.

**Abbreviations:** HU: Haunsfield’s unit NIHSS: The National Institutes of Health Stroke Scale, CT: Brain computed tomography
